
JOURNAL INFORMATION
==============================
NLM Title Abbreviation: ScientificWorldJournal
Journal ID: ScientificWorldJournal
NLM Journal ID: 101131163
Journal ID: TSWJ

The Scientific World Journal

ISSN: 2356-6140
EISSN: 1537-744X
Publisher: Wiley

ARTICLE INFORMATION
==============================
PMCID: PMC6084131
PMID: 30147555
DOI: 10.1100/tsw.2001.113
Article ID: 496421
Article version: 1
Subjects: Short Report

Delaying Aging with Mitochondrial Micronutrients and Antioxidants

Ames Bruce N. *
Liu Jiankang
Atamna Hani
University of California, Berkeley/CHORI 5700, MLK Jr Way, Oakland, CA 96409, USA
*Bruce N. Ames: bames@chori.org
Publication date: 2001
Electronic publication date: 2001 Sep 24
Volume: 1
First page: 81
Last page: 82
Copyright: Copyright © 2001 Hani Atamna et al.
Copyright year: 2001
License: This is an open access article distributed under the Creative Commons Attribution License, which permits unrestricted use, distribution, and reproduction in any medium, provided the original work is properly cited.
License URL: https://creativecommons.org/licenses/by/3.0/

==============================
Miami *Nature Biotechnology* Short Reports
*TheScientificWorld* (2001) 1(S3), 81-82SR
ISSN 1532-2246; DOI 10.1100/TSW.2001.113

### DELAYING AGING WITH MITOCHONDRIAL MICRONUTRIENTS AND ANTIOXIDANTS

Bruce N. Ames*, Jiankang Liu and Hani Atamna

University of California, Berkeley/CHORI 5700, MLK Jr Way, Oakland, CA 96409
* Bames@chori.org

**MITOCHONDRIAL DECAY IN AGING.** Mitochondria decay with age due to the oxidation of mtDNA, proteins, and lipids. Some of this decay can be reversed in old rats by feeding them normal mitochondrial metabolites at high levels. Aging appears to be in good part due to the oxidants produced as by-products of normal metabolism by mitochondria [1-5]. In old rats mitochondrial membrane potential, cardiolipin levels, respiratory control ratio, and overall cellular O2 consumption are lower than in young rats and the level of oxidants (per unit O2) and mutagenic aldehydes from lipid peroxidation is higher [3]. Ambulatory activity declines markedly in old rats. Spatial memory as measured by the Morris water maze, also declines with age. Feeding old rats the normal mitochondrial metabolites acetyl carnitine and lipoic acid for a few weeks, restores mitochondrial function, lowers oxidants to the level of a young rat, and increases ambulatory activity and spatial memory [6-11]. Thus, these two metabolites can be considered necessary for health in old age and are therefore “conditional micronutrients”. This restoration suggests a plausible mechanism [11]: with age increased oxidative damage to proteins, such as acyl carnitine transferase (whose activity declines with age), and lipid membranes, particularly in mitochondria, causes a deformation of structure of key enzymes, with a consequent lessening of affinity (Km) for the enzyme substrate; an increased level of the substrate restores the velocity of the reaction, and thus restores function.

Hepatocytes were isolated from young (3-5 months) and old (24-28 months) rats and incubated with various concentrations of *tert*-butylhydroperoxide (t-BuOOH) [10]. The t-BuOOH concentration that killed 50% of cells (LC50) in 2 hr declined nearly two-fold from 721 ± 32 $\mu M$ in cells from young rats to 391 ± 31 $\mu M$ in cells from old rats. This increased sensitivity of hepatocytes from old rats may be due, in part, to changes in glutathione (GSH) levels, because total cellular and mitochondrial GSH were 37.7% and 58.3% lower, respectively, compared to cells from young rats. Cells from old animals were incubated with either (*R*)- or (*S*)-lipoic acid (100 $\mu M$) for 30 min prior to the addition of 300 $\mu M$ t-BuOOH. The physiologically relevant (*R*)-form, a coenzyme in mitochondria, as opposed to the (*S*)-form significantly protected hepatocytes against t-BuOOH toxicity. Dietary supplementation of (*R*)-lipoic acid [0.5% (wt/wt)] for 2 weeks also completely reversed the age-related decline in hepatocellular GSH levels and the increased vulnerability to t-BuOOH as well. An identical supplemental diet fed to young rats did not enhance the resistance to t-BuOOH, indicating that antioxidant protection was already optimal in young rats. Thus, this study shows that cells from old animals are more susceptible to oxidant insult and (*R*)-lipoic acid, after reduction to an antioxidant in the mitochondria, effectively reverses this age-related increase in oxidant vulnerability.

A dose-response study has been done on ALCAR [Liu & Ames, in preparation]. Previously we showed that acetyl-L-carnitine (ALCAR) fed to old rats (1.5% in drinking water) partially restored the age-associated decline in liver mitochondrial function and ambulatory activity but increased oxidative stress [6]. Aged rats (23 months old) were given ALCAR at 0.15%, 0.5%, and 1.5% in drinking water for 4 weeks. Lower concentrations of ALCAR, both 0.15% and 0.5% concentrations, increased the free, acyl, and total carnitines in the brain and plasma to the levels of untreated young rats as well as the 1.5% concentration. In addition, lower concentrations of ALCAR (both 0.15% and 0.5%) reversed the age-associated decline in ambulatory activity and mitochondrial cristae loss in the dentate gyrus of the hippocampus more significantly than the 1.5% dose. The lower doses had no effect on protein oxidation, in contrast to the 1.5% dose, which caused an increase in protein carbonyls in the brain. The lower doses appeared to reduce the age-dependent increase in malondialdehyde, an end product of lipid peroxidation. These results suggest: 1) that causing oxidative stress, a side effect of ALCAR, may only occur at very high dose to old rats, and 2) a lower dose of ALCAR administration can improve brain function by reversing the age-associated mitochondrial decay, by repairing mitochondrial structure, and possibly by reducing oxidative stress.

We have now extended our previous study of ALCAR and lipoic acid on mitochondrial function in liver of old rats to brain function. We examined the effects in old rats of lower doses of ALCAR (0.2% and 0.5%) and lipoic acid (0.1% and 0.2%) as well as their combination on spatial memory with the Morris water maze test [Liu & Ames, in preparation] and on implicit memory with a Skinner box test [Gharib *et al.*, in preparation]. Both ALCAR and lipoic acid showed effects in improving the age-associated memory decline, however, the combination showed a more significant improvement in both memory tests.

**PBN.** Before showing that lipoic acid reversed oxidative mitochondrial decay we showed that α-phenyl-N-*t*-butyl nitrone (PBN), a widely used spin trapping agent, was effective in lowering oxidative damage in old rats and that it was effective with acetyl carnitine [9]. We had previously shown that PBN delays the senescence of human diploid fibroblasts (IMR90 cells) in culture [12]. PBN reverses the age-related oxidative changes in the brains of old gerbils [13, 14] and delays senescence in senescence-accelerated mice [15] and in normal mice [16]. N-*t*-butyl hydroxylamine (NtBHA) and benzaldehyde are the breakdown products of PBN [17]. N-*t*-butyl hydroxylamine delays senescence of IMR90 cells at concentrations as low as 10 µM compared to 200 µM of PBN to produce a similar effect, suggesting that N-*t*-butyl hydroxylamine is the active form of PBN. N-Benzyl hydroxylamine and N-methyl hydroxylamine, compounds unrelated to PBN, were also effective in delaying senescence, suggesting the active functional group is the N-hydroxylamine. All the N-hydroxylamines tested significantly decreased the endogenous production of oxidants, as measured by the oxidation of 2′,7′-dichlorodihydrofluorescin (DCFH), and increased the GSH/GSSG ratio. The acceleration of senescence induced by hydrogen peroxide is reversed by the N-hydroxylamines. DNA damage, as determined by the level of apurinic/apyrimidinic (AP) sites, also decreased significantly following treatment with N-hydroxylamines. The N-hydroxylamines appear to be effective through mitochondria: they delay age-dependent changes in mitochondria as measured by accumulation of rhodamine-123; they prevent reduction of cytochrome $C^{III}$ by superoxide radical; and they reverse an age-dependent decay of mitochondrial aconitase, suggesting they react with the superoxide radical.

In a second study [18] the effect of NtBHA on mitochondria in old and young rats and human primary fibroblasts (IMR90) was examined. In NtBHA treated rats, the age-dependent decline in food consumption and ambulatory activity was reversed, without affecting body weight. The respiratory control ratio (RCR) of mitochondria from liver was greater, indicating that mitochondria from treated rats were more coupled. NtBHA, also reversed the age-dependent decline in the activity of glutamate dehydrogenase, a mitochondrial enzyme, but had no effect on the cytosolic enzyme glucose-6-phosphate dehydrogenase. These findings suggest that NtBHA improved mitochondrial function *in vivo*. The age-dependent increase in proteins with thiol-mixed disulfides was significantly lower in old rats treated with NtBHA. NtBHA was effective in improving mitochondrial function only in old rats; no effect was observed in young rats. In IMR90 cells, NtBHA delayed senescence-associated changes to mitochondria and cellular senescence that was induced by maintaining the cells under sub-optimal levels of growth factors.

Proteasomal activity was also higher in cells treated with NtBHA than untreated cells. Mechanistic studies using IMR90 cells suggest that complex III and cytochrome c are the mitochondrial components that interact with NtBHA.

**OXIDATIVE MUTAGENS.** Mitochondrial decay during aging produces oxidative mutagens such as hydrogen peroxide [3] and mutagenic products of lipid peroxidation such as enals [19] and alkylating agents. We have developed a method for measuring AP (apurinic/apyrimidinic) sites in the DNA of living cells produced from oxidants or alkylating agents [20]. The number of AP sites in old IMR90 cells was about two to three times higher than that in young cells, and the number in human leukocytes from old donors was about seven times that in young donors. The repair of AP sites was slower in senescent compared with IMR90 cells. An age-dependent decline is shown in the activity of the gylcosylase that removes oxidized bases in IMR90 cells and alkylated bases in human leukocytes. NtBHA decreased the age-associated increase in AP sites in IMR90 cells suggesting they are due to oxidants leaking from mitochondria [17].

**REFERENCES.**

1. Shigenaga, M.K., Hagen, T.M., and Ames, B.N. (1994) Proc. Natl. Acad. Sci. U S A 91, 10771-10778
2. Beckman, K.B. and Ames, B.N. (1998) Physiol. Rev. 78, 547-581
3. Hagen, T.M. et al. (1997) Proc. Natl. Acad. Sci. U S A 94, 3064-3069
4. Helbock, H.J. et al. (1998) Proc. Natl. Acad. Sci. U S A 95, 288-293
5. Beckman, K.B. and Ames, B.N. (1998) Ann. N. Y. Acad. Sci. 854, 118-127
6. Hagen, T.M. et al. (1998) Proc. Natl. Acad. Sci. U S A 95, 9562-9566
7. Lykkesfeldt, J. et al. (1998) FASEB J. 12, 1183-1189
8. Hagen, T.M. et al. (1998) FASEB J. 13, 411-418
9. Hagen, T.M., Wehr, C.M., and Ames, B.N. (1998) Ann. N. Y. Acad. Sci. 854, 214-223
10. Hagen, T.M. et al. (2000) Antiox. Redox Signal. 2, 473-483
11. Ames, B.N. (1998) Toxicol. Lett. 102-103: p. 5-18.
12. Chen, Q. et al. (1995) Proc. Natl. Acad. Sci. U S A 92, 4337-4341
13. Oliver, C.N. et al. (1990) Proc. Natl. Acad. Sci. U S A 87, 5144-5147
14. Carney, J.M. et al. (1991) Proc. Natl. Acad. Sci. U S A 88(9), 3633-3636

15. Edamatsu, R., Mori, A., and Packer, L. (1995) Biochem. Biophys. Res. Commun. 211(3), 847-849
16. Saito, K., Yoshioka, H., and Cutler, R.G. (1998) Biosci. Biotechnol. Biochem. 62(4), 792-794
17. Atamna, H., Paler-Martinez, A., and Ames, B.N. (2000) J. Biol. Chem. 275(10), 6741-6748
18. Atamna, H. et al. (2000) J. Biol. Chem., submitted.
19. Marnett, L.J. et al. (1985) Mutat. Res. 148, 25-34
20. Atamna, H., Cheung, I., and Ames, B.N. (2000) Proc. Natl. Acad. Sci. U S A 97(2), 686-691
